# Erratum: Conducting clinical research in the era of the COVID-19 pandemic: Challenges and lessons for Speech-Language Pathology and Audiology research

**DOI:** 10.4102/sajcd.v70i1.942

**Published:** 2023-05-23

**Authors:** Katijah Khoza-Shangase, Nomfundo Moroe, Ben Sebothoma

**Affiliations:** 1 Department of Audiology, Faculty of Humanities, University of the Witwatersrand, Johannesburg, South Africa

In the published article, Khoza-Shangase, K., Monroe, N., & Sebothoma, B. (2022). Conducting clinical research in the era of the COVID-19 pandemic: Challenges and lessons for Speech-Language Pathology and Audiology research. *South African Journal of Communication Disorders, 69*(2), a898. https://doi.org/10.4102/sajcd.v69i2.898, an author name was incorrectly spelt as Monroe. The correct spelling is Moroe.

The publisher apologises for this error. The correction does not change the study’s findings of significance or overall interpretation of the study’s results or the scientific conclusions of the article in any way.

